# Supplementary material for: End Sequence Analysis Toolkit (ESAT) expands the extractable information from single-cell RNA-seq data
Source: Genome Res. 2016 Oct;26(10):1397–410. doi: 10.1101/gr.207902.116 (PMC5052061; doi:10.1101/gr.207902.116)
Supplement: Supplemental Material [file supp_gr.207902.116_Supp_Materials.tar.gz › ESAT-esat_v0.1/doc/html/classbroad_1_1core_1_1parser_1_1_command_line_parser.html]

ESAT: broad.core.parser.CommandLineParser Class Reference


ESAT


- Main Page
- Related Pages
- Packages
- Classes
- Files

- Class List
- Class Index
- Class Hierarchy
- Class Members

All Classes Namespaces Files Functions Variables Enumerator Pages

- broad
- core
- parser
- CommandLineParser

Public Member Functions |
List of all members

broad.core.parser.CommandLineParser Class Reference

|  |  |
| --- | --- |
| Public Member Functions | |
|  | CommandLineParser () |
|  | |
| void | setProgramDescription (String description) |
|  | |
| void | addStringArg (String flag, String description, boolean required) |
|  | |
| void | addStringArg (String flag, String description, boolean required, String def) |
|  | |
| void | addStringListArg (String flag, String description, boolean required, ArrayList< String > def) |
|  | |
| void | addStringListArg (String flag, String description, boolean required) |
|  | |
| void | addIntArg (String flag, String description, boolean required) |
|  | |
| void | addIntArg (String flag, String description, boolean required, int def) |
|  | |
| void | addFloatArg (String flag, String description, boolean required) |
|  | |
| void | addFloatArg (String flag, String description, boolean required, float def) |
|  | |
| void | addDoubleArg (String flag, String description, boolean required) |
|  | |
| void | addDoubleArg (String flag, String description, boolean required, double def) |
|  | |
| void | addBooleanArg (String flag, String description, boolean required) |
|  | |
| void | addBooleanArg (String flag, String description, boolean required, boolean def) |
|  | |
| void | parse (String[] args, boolean allowDuplicateTags) |
|  | |
| void | parse (String[] args) |
|  | |
| Map< String, String > | getFlagsAndValues () |
|  | |
| String | getArgString () |
|  | |
| String | getArgString (Collection< String > flagsToRemove) |
|  | |
| String | getStringArg (String flag) |
|  | |
| ArrayList< String > | getStringListArg (String flag) |
|  | |
| int | getIntArg (String flag) |
|  | |
| float | getFloatArg (String flag) |
|  | |
| double | getDoubleArg (String flag) |
|  | |
| boolean | getBooleanArg (String flag) |
|  | |
| void | printHelpMessage () |
|  | |
| boolean | hasIntFlag (String flag) |
|  | |
| boolean | hasFloatFlag (String flag) |
|  | |
| boolean | hasDoubleFlag (String flag) |
|  | |
| boolean | hasBooleanFlag (String flag) |
|  | |
| boolean | hasStringFlag (String flag) |
|  | |

## Detailed Description

Author
:   prussell Full-featured command line parser

## Constructor & Destructor Documentation

|  |  |  |  |  |
| --- | --- | --- | --- | --- |
| broad.core.parser.CommandLineParser.CommandLineParser | ( |  | ) |  |

## Member Function Documentation

|  |  |  |  |
| --- | --- | --- | --- |
| void broad.core.parser.CommandLineParser.addBooleanArg | ( | String | *flag*, |
|  |  | String | *description*, |
|  |  | boolean | *required* |
|  | ) |  |  |

Adds new boolean argument to set of arguments Client will crash if the same argument flag or description has already been added

Parameters
:   |  |  |
    | --- | --- |
    | flag | the command line flag for the argument |
    | description | the description of the argument |
    | required | whether parameter is required |

|  |  |  |  |
| --- | --- | --- | --- |
| void broad.core.parser.CommandLineParser.addBooleanArg | ( | String | *flag*, |
|  |  | String | *description*, |
|  |  | boolean | *required*, |
|  |  | boolean | *def* |
|  | ) |  |  |

Adds new boolean argument to set of arguments and stores default Client will crash if the same argument flag or description has already been added

Parameters
:   |  |  |
    | --- | --- |
    | flag | the command line flag for the argument |
    | description | the description of the argument |
    | required | whether parameter is required |
    | def | default value |

|  |  |  |  |
| --- | --- | --- | --- |
| void broad.core.parser.CommandLineParser.addDoubleArg | ( | String | *flag*, |
|  |  | String | *description*, |
|  |  | boolean | *required* |
|  | ) |  |  |

Adds new double argument to set of arguments Client will crash if the same argument flag or description has already been added

Parameters
:   |  |  |
    | --- | --- |
    | flag | the command line flag for the argument |
    | description | the description of the argument |
    | required | whether parameter is required |

|  |  |  |  |
| --- | --- | --- | --- |
| void broad.core.parser.CommandLineParser.addDoubleArg | ( | String | *flag*, |
|  |  | String | *description*, |
|  |  | boolean | *required*, |
|  |  | double | *def* |
|  | ) |  |  |

Adds new double argument to set of arguments and stores default Client will crash if the same argument flag or description has already been added

Parameters
:   |  |  |
    | --- | --- |
    | flag | the command line flag for the argument |
    | description | the description of the argument |
    | required | whether parameter is required |
    | def | default value |

|  |  |  |  |
| --- | --- | --- | --- |
| void broad.core.parser.CommandLineParser.addFloatArg | ( | String | *flag*, |
|  |  | String | *description*, |
|  |  | boolean | *required* |
|  | ) |  |  |

Adds new float argument to set of arguments Client will crash if the same argument flag or description has already been added

Parameters
:   |  |  |
    | --- | --- |
    | flag | the command line flag for the argument |
    | description | the description of the argument |
    | required | whether parameter is required |

|  |  |  |  |
| --- | --- | --- | --- |
| void broad.core.parser.CommandLineParser.addFloatArg | ( | String | *flag*, |
|  |  | String | *description*, |
|  |  | boolean | *required*, |
|  |  | float | *def* |
|  | ) |  |  |

Adds new float argument to set of arguments and stores default Client will crash if the same argument flag or description has already been added

Parameters
:   |  |  |
    | --- | --- |
    | flag | the command line flag for the argument |
    | description | the description of the argument |
    | required | whether parameter is required |
    | def | default value |

|  |  |  |  |
| --- | --- | --- | --- |
| void broad.core.parser.CommandLineParser.addIntArg | ( | String | *flag*, |
|  |  | String | *description*, |
|  |  | boolean | *required* |
|  | ) |  |  |

Adds new int argument to set of arguments Client will crash if the same argument flag or description has already been added

Parameters
:   |  |  |
    | --- | --- |
    | flag | the command line flag for the argument |
    | description | the description of the argument |
    | required | whether parameter is required |

|  |  |  |  |
| --- | --- | --- | --- |
| void broad.core.parser.CommandLineParser.addIntArg | ( | String | *flag*, |
|  |  | String | *description*, |
|  |  | boolean | *required*, |
|  |  | int | *def* |
|  | ) |  |  |

Adds new int argument to set of arguments and stores default Client will crash if the same argument flag or description has already been added

Parameters
:   |  |  |
    | --- | --- |
    | flag | the command line flag for the argument |
    | description | the description of the argument |
    | required | whether parameter is required |
    | def | default value |

|  |  |  |  |
| --- | --- | --- | --- |
| void broad.core.parser.CommandLineParser.addStringArg | ( | String | *flag*, |
|  |  | String | *description*, |
|  |  | boolean | *required* |
|  | ) |  |  |

Adds new string argument to set of arguments Client will crash if the same argument flag or description has already been added

Parameters
:   |  |  |
    | --- | --- |
    | flag | the command line flag for the argument |
    | description | the description of the argument |
    | required | whether parameter is required |

|  |  |  |  |
| --- | --- | --- | --- |
| void broad.core.parser.CommandLineParser.addStringArg | ( | String | *flag*, |
|  |  | String | *description*, |
|  |  | boolean | *required*, |
|  |  | String | *def* |
|  | ) |  |  |

Adds new string argument to set of arguments and stores default Client will crash if the same argument flag or description has already been added

Parameters
:   |  |  |
    | --- | --- |
    | flag | the command line flag for the argument |
    | description | the description of the argument |
    | required | whether parameter is required |
    | def | default value |

|  |  |  |  |
| --- | --- | --- | --- |
| void broad.core.parser.CommandLineParser.addStringListArg | ( | String | *flag*, |
|  |  | String | *description*, |
|  |  | boolean | *required*, |
|  |  | ArrayList< String > | *def* |
|  | ) |  |  |

Adds new string list argument to set of arguments and stores default Client will crash if the same argument flag or description has already been added

Parameters
:   |  |  |
    | --- | --- |
    | flag | the command line flag for the argument |
    | description | the description of the argument |
    | required | whether parameter is required |
    | def | default value |

|  |  |  |  |
| --- | --- | --- | --- |
| void broad.core.parser.CommandLineParser.addStringListArg | ( | String | *flag*, |
|  |  | String | *description*, |
|  |  | boolean | *required* |
|  | ) |  |  |

Adds new string list argument to set of arguments and stores default Client will crash if the same argument flag or description has already been added

Parameters
:   |  |  |
    | --- | --- |
    | flag | the command line flag for the argument |
    | description | the description of the argument |
    | required | whether parameter is required |
    | def | default value |

|  |  |  |  |  |
| --- | --- | --- | --- | --- |
| String broad.core.parser.CommandLineParser.getArgString | ( |  | ) |  |

Get the argument string from the command line

Returns
:   The argument string

|  |  |  |  |  |  |
| --- | --- | --- | --- | --- | --- |
| String broad.core.parser.CommandLineParser.getArgString | ( | Collection< String > | *flagsToRemove* | ) |  |

Get the argument string from the command line, possibly leaving out some arguments

Parameters
:   |  |  |
    | --- | --- |
    | flagsToRemove | Flags to leave out |

Returns
:   The argument string

Here is the call graph for this function:

|  |  |  |  |  |  |
| --- | --- | --- | --- | --- | --- |
| boolean broad.core.parser.CommandLineParser.getBooleanArg | ( | String | *flag* | ) |  |

Get value of boolean parameter specified by flag

Parameters
:   |  |  |
    | --- | --- |
    | flag | The command line flag for the argument |

Returns
:   Boolean specified on command line or null if parameter was not specified

Here is the caller graph for this function:

|  |  |  |  |  |  |
| --- | --- | --- | --- | --- | --- |
| double broad.core.parser.CommandLineParser.getDoubleArg | ( | String | *flag* | ) |  |

Get value of double parameter specified by flag

Parameters
:   |  |  |
    | --- | --- |
    | flag | The command line flag for the argument |

Returns
:   Double specified on command line or null if parameter was not specified

|  |  |  |  |  |
| --- | --- | --- | --- | --- |
| Map<String, String> broad.core.parser.CommandLineParser.getFlagsAndValues | ( |  | ) |  |

Get the flags and values that were specified on the command line

Returns
:   Map of flag to value

Here is the caller graph for this function:

|  |  |  |  |  |  |
| --- | --- | --- | --- | --- | --- |
| float broad.core.parser.CommandLineParser.getFloatArg | ( | String | *flag* | ) |  |

Get value of float parameter specified by flag

Parameters
:   |  |  |
    | --- | --- |
    | flag | The command line flag for the argument |

Returns
:   Float specified on command line or null if parameter was not specified

|  |  |  |  |  |  |
| --- | --- | --- | --- | --- | --- |
| int broad.core.parser.CommandLineParser.getIntArg | ( | String | *flag* | ) |  |

Get value of int parameter specified by flag

Parameters
:   |  |  |
    | --- | --- |
    | flag | The command line flag for the argument |

Returns
:   Integer specified on command line or null if parameter was not specified

|  |  |  |  |  |  |
| --- | --- | --- | --- | --- | --- |
| String broad.core.parser.CommandLineParser.getStringArg | ( | String | *flag* | ) |  |

Get value of String parameter specified by flag

Parameters
:   |  |  |
    | --- | --- |
    | flag | The command line flag for the argument |

Returns
:   String specified on command line or null if parameter was not specified

Here is the caller graph for this function:

|  |  |  |  |  |  |
| --- | --- | --- | --- | --- | --- |
| ArrayList<String> broad.core.parser.CommandLineParser.getStringListArg | ( | String | *flag* | ) |  |

Get value of String parameter specified by flag

Parameters
:   |  |  |
    | --- | --- |
    | flag | The command line flag for the argument |

Returns
:   String specified on command line or null if parameter was not specified

|  |  |  |  |  |  |
| --- | --- | --- | --- | --- | --- |
| boolean broad.core.parser.CommandLineParser.hasBooleanFlag | ( | String | *flag* | ) |  |

Whether the flag is associated with a boolean argument and was specified on the command line

Parameters
:   |  |  |
    | --- | --- |
    | flag | The flag |

Returns
:   Whether the flag is present

|  |  |  |  |  |  |
| --- | --- | --- | --- | --- | --- |
| boolean broad.core.parser.CommandLineParser.hasDoubleFlag | ( | String | *flag* | ) |  |

Whether the flag is associated with a double argument and was specified on the command line

Parameters
:   |  |  |
    | --- | --- |
    | flag | The flag |

Returns
:   Whether the flag is present

|  |  |  |  |  |  |
| --- | --- | --- | --- | --- | --- |
| boolean broad.core.parser.CommandLineParser.hasFloatFlag | ( | String | *flag* | ) |  |

Whether the flag is associated with a float argument and was specified on the command line

Parameters
:   |  |  |
    | --- | --- |
    | flag | The flag |

Returns
:   Whether the flag is present

|  |  |  |  |  |  |
| --- | --- | --- | --- | --- | --- |
| boolean broad.core.parser.CommandLineParser.hasIntFlag | ( | String | *flag* | ) |  |

Whether the flag is associated with an int argument and was specified on the command line

Parameters
:   |  |  |
    | --- | --- |
    | flag | The flag |

Returns
:   Whether the flag is present

|  |  |  |  |  |  |
| --- | --- | --- | --- | --- | --- |
| boolean broad.core.parser.CommandLineParser.hasStringFlag | ( | String | *flag* | ) |  |

Whether the flag is associated with a string argument and was specified on the command line

Parameters
:   |  |  |
    | --- | --- |
    | flag | The flag |

Returns
:   Whether the flag is present

|  |  |  |  |
| --- | --- | --- | --- |
| void broad.core.parser.CommandLineParser.parse | ( | String[] | *args*, |
|  |  | boolean | *allowDuplicateTags* |
|  | ) |  |  |

Parse command arguments If command line is not in proper form, prints help menu and exits If a required argument is missing, prints help menu and exits

Parameters
:   |  |  |
    | --- | --- |
    | args | the command line arguments passed to a main program |

Here is the call graph for this function:

Here is the caller graph for this function:

|  |  |  |  |  |  |
| --- | --- | --- | --- | --- | --- |
| void broad.core.parser.CommandLineParser.parse | ( | String[] | *args* | ) |  |

Here is the call graph for this function:

|  |  |  |  |  |
| --- | --- | --- | --- | --- |
| void broad.core.parser.CommandLineParser.printHelpMessage | ( |  | ) |  |

Prints program description plus argument flags and descriptions

Here is the caller graph for this function:

|  |  |  |  |  |  |
| --- | --- | --- | --- | --- | --- |
| void broad.core.parser.CommandLineParser.setProgramDescription | ( | String | *description* | ) |  |

Sets program description to be printed as part of help menu

Parameters
:   |  |  |
    | --- | --- |
    | description | The program description |

---

The documentation for this class was generated from the following file:

- java/broad/core/parser/CommandLineParser.java


---

Generated on Fri Aug 15 2014 15:10:55 for ESAT by  

 1.8.7
